# Sarcoidosis with heart involvement: a rare association of terrible prognosis, a report of two cases

**DOI:** 10.11604/pamj.2015.21.243.6124

**Published:** 2015-08-05

**Authors:** Asma Kefi, Nadia Ben Abdelhafidh, Sameh Sayhi, Rim Abid, Faida Ajili, Janet Laabidi, Salah Othmani

**Affiliations:** 1 Department of Internal Medicine, Military Hospital of Tunis, 1008 Montfleury, Tunisia

**Keywords:** Cardiac sarcoidosis, Sarcoidosis, Ventricular arrhythmias

## Abstract

Sarcoidosis is a multisystemic disorder of unknown etiology which is characterized by the formation of non-caseating granulomas in involved tissues. Cardiac involvement is one of the least common manifestations and it can occur at any point of time during the course of sarcoidosis. Here we present the case of 2 patients with known sarcoidosis who develop cardiac abnormalities in the absence of known primary cardiac cause. In our report, we would like to draw attention to the importance of considering heart involvement in any case with systemic sarcoidosis especially in young age.

## Introduction

Sarcoidosis, an enigmatic multisystemic disease, is characterized by the formation of granulomas in many tissues mainly the lungs, the lymphoreticular system, the eyes, and the skin, with cardiac involvement being a rare entity [1]. Many reports showed that the quoted incidence of heart involvement in sarcoidosis in their review was around 2%, so that it was one of the least common manifestations [1]. The clinical sequelae of cardiac sarcoidosis vary from asymptomatic conduction abnormalities to heart failure, fatal ventricular arrhythmias, and sudden death. Foregoing data, early diagnosis and prompt treatment are essential since they can improve prognosis [2] by reducing the chances of sudden death.

## Patient and observation

### Case 1

**Figure 1:**
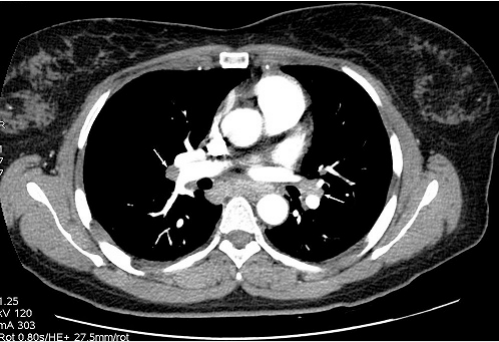
Cardiac magnetic resonance imaging (MRI) with gadolinium contrast: evidence of delayed hyper enhancement (arrow) in both the basal and inferolateral left ventricular regions

**Figure 2:**
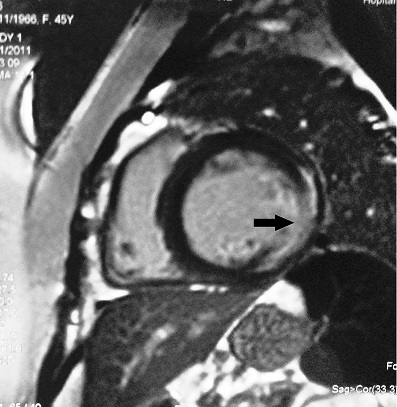
Decreased uptake in the inferolateral and apical ventricular myocardium, which was reversible during stress (reverse distribution) shown on Technetium99m sestamibiscintigraphy myocardial perfusion study

A 40-year-old Tunisian woman, with a history of diabetes mellitus in diet and Lofgren's syndrome (an acute presentation consisting of polyarthralgia, erythema nodosum, bilateral intrathoracic lymphadenopathies) diagnosed in February 2013, came to our department, in February 2014, for exploration of exertional dyspnea of one month evolution and progressively worsening without fever, chest pain, or syncope. The patient denied any past history of hypertension, coronary artery, or thyroid disease. She did not smoke or consume alcohol. On physical examination, she was conscious, cooperative, and afebrile. She was hemodynamically stable (blood pressure (BP) 130/70 mmHg and had a regular pulse of 88 beats /minute.). She had good color and was slightly polypneic with no peripheral cyanosis (peripheral oxygen saturation 97%). She presented no palpable adenopathy or signs of jugular distension. Cardiac auscultation revealed rhythmic sounds with no murmur or friction rub. There were no signs of peripheral edema or deep vein thrombosis. Pulmonary, abdominal, and neurological examination revealed no abnormalities. The admission electrocardiogram (ECG) was normal. On investigation, blood biochemistry, liver, renal function, thyroid function and, hemogram showed no abnormalities except lymphopenia. Her angiotensin converting enzyme (ACE) level was 132 units (normal range < 68units). The immunological markers were negative. A contrast-enhanced computed tomography (CT) chest scan showed almost complete regression of mediastinal lymphadenopathies without pleuroparenchymal lesion. Respiratory functional tests and bronchoscopy with transbronchial lung biopsy were normal. The transthoracic echocardiography with Doppler showed abnormal left ventricular relaxation and systolic dysfunction of the left ventricle with ventricular ejection fraction (EF) of 40%. In the light of these symptoms and echocardiographic abnormalities, for which other possible causes have been excluded, the diagnosis of sarcoidosis with heart involvement was suspected. Thus, more specialized cardiac evaluation was needed. 24 hour holter monitoring showed recurrent episodes of 2000 monomorphic ventricular extrasystoles. Cardiac magnetic resonance imaging (MRI) with gadolinium contrast showed evidence of delayed hyper enhancement in both the basal and inferolateral left ventricular regions, suspicious for fibrogranulomatous tissue of sarcoidosis (Figure 1). The left ventricular ejection fraction was 40%. Laboratory tests confirmed negative serial markers of myocardial necrosis. Technetium99m sestamibi scintigraphy myocardial perfusion study showed decreased uptake in the inferolateral and apical ventricular myocardium, which was reversible during stress (reverse distribution) (Figure 2). Coronary angiography and let ventriculography showed angiographically normal coronary arteries. Considering all these clinical and investigatioanal findings in a patient with a history of Lofgren's syndrome and in the absence of primary heart disease, the diagnosis of cardiac sarcoidosis was made. The patient started with a dose of 1mg/kg/day of corticosteroids in addition to supportive care. This dose was tapered gradually to a maintenance level of 15 mg per day over 5 months. To this day, evolution was favorable.

### Case 2

**Figure 3:**
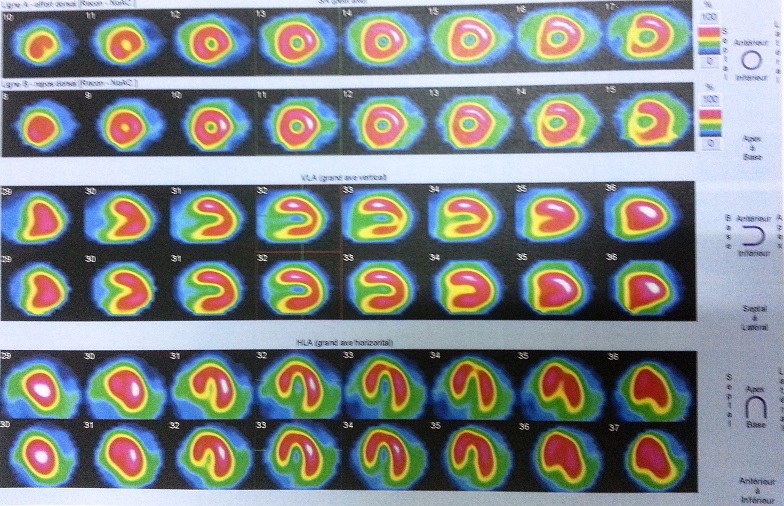
High-resolution computed tomography scan of the chest showing peribronchial and bilateral hilar lymphadenopathy (arrows)

A 48-year-old Tunisian woman, presented to our consultations with a history of intermittent palpitations and exertional dyspnea of 4 months duration, progressively worsening for 2 weeks before, without any history of chest pain or syncope. She had a past history of systemic sarcoidosis diagnosed in April 2009 on the basis of the following compatible clinical and radiologic findings: systemic symptoms (such as fatigue, anorexia, and weight loss), parotid enlargement, negative tuberculin skin test, lymphopenia, hypercalciuria, increased serum ACE level, intrathoracic lymphadenopathies which were hilar (Figure 3), bilateral symmetrical, and associated with right paratracheal window lymph node involvement. Pulmonary functions tests demonstrated decreased volumes and CO diffusing capacity. Lymphocytosis in broncho-alveolar lavage was observed and a CD4+/CD8+ lymphocyte ratio was greater than 3.5. Lip biopsy of accessory salivary glands was compatible with grade 1 according to Chisholm's classification. After ruling out the other granulomatous diseases such as tuberculosis, lymphoma, crohn's disease, Wegner granulomatosis…, the diagnosis of systemic sarcoisosis was ascertained. A short-course corticosteroid therapy with antimalarial drug were initiated with a favorable evolution both clinically and radiogically. The patient was presented in November 2011 with a history of intermittent palpitations and exertionaldyspnea of 4 months duration, progressively worsening for 2 weeks before. She denied any history of hypertension, diabetes mellitus, coronary artery disease and thyroid disease. She did not smoke, consume alcohol, or illicit drugs. On physical examination, she was conscious, cooperative, and afebrile. She had a BP of 120/70 mmHg and a pulse of 160 beats/minute. There were no signs of cardiac failure; the rest of the physical examination did not reveal any abnormal findings. She was admitted to the cardiac intensive care unit. The admission ECG showed a ventricular tachycardia at 160 cycles/minute. In the light of these typical symptoms and electrocardiographic alterations in a patient having systematic sarcoidosis, the diagnosis of sarcoidosis with cardiac involvement was suspected. The echocardiography showed a FE of 50%, bright shadows consisting with infiltration in the basal and inferoseptal ventricular myocardium, and moderate mitral regurgitation. Coronary angiography revealed normal coronary arteries. The cardiac electrophysiology study showed a monomorphic ventricular tachycardia induced by isoproterenol. Laboratory tests showed no relevant alterations in myocardial necrosis biomarkers, blood cell count, renal and liver function, or electrolytes. Given the absence of primary heart disease that would explain the cardiac abnormalities and especially the previous diagnosis of systemic sarcoidosis, the final diagnosis was sarcoidosis with cardiac involvement. The patient started with a dose of 1 mg/kg/day of corticosteroid, in addition to supportive care. Over 8 months, the steroids were tapered to a dose of 10 mg/day. Anti arrhythmic drug therapy (Amiodarone) and β blocker were initiated. Given the recurrent ventricular tachycardia with amiodarone, we proceeded to the implantation of an automated implantable cardioverter-defibrillator (ICD). Evolution was favorable.

## Discussion

Sarcoidosis is a multisystemic disorder of unknown etiology which is characterized by the formation of non-caseating granulomas in involved tissues. To this day, it remains something of enigma, the search for an etiologic agent is ongoing [2]. Sarcoidosis can be widespread or limited to involvement of only a single system at a time, it most commonly involves the lungs, the lymph nodes of the thorax and the eye, despite the fact that virtually no organ systems are immune to infiltration by sarcoid granulomas. Cardiac involvement is one of the least common manifestations and it can occur at any point of time during the course of sarcoidosis. Vishal S et al reported that the incidence of clinical heart involvement was as approximately 5%, wheras at autopsy, the incidence was considerably higher (20 to 25%) [2].

Making a diagnosis of cardiac sarcoidosis remains one of the main challenges in the management of the disease: in fact, if cardiac manifestations occur in patients with multisystemic sarcoidosis, the diagnosis, although circumstancial, is strongly suspected, which is in keeping with our 2 cases. However, when cardiac dysfunction is the only manifestation of sarcoidosis, the diagnosis is frequently not entertained. Clinical manifestations of cardiac sarcoidosis, ranging from asymptomatic conduction abnormalities to fatal ventricular arrhythmia or sudden death, are dependent on both the profusion and location of granulomas. Granulomas can involve the left ventricular free wall, basal ventricular septum, right ventricle, papillary muscle, right atrium, and left atrium [3]. Main relevant signs include complete heart block, ventricular tachycardia, supra ventricular arrhythmias (atrial flutter, fibrillation and paroxysmal atrial tachycardia), congestive heart failure, and sudden death. Other clinical manifestations may involve chest pain, electrocardiographic changes, and pericardial abnormalities (pericardial effusion, constrictive pericarditis, tamponade) [2]. Despite the fact that endomyocardial biopsy is theoretically the most confident mean to assert the diagnosis of cardiac sarcoidosis, it is an invasive procedure which lacks sensitivity: cardiac involvement tends to be patchy and granulomas are more likely to be located in the left ventricle and basal ventricular septum than in the right ventricle where endomyocardial biopsies are usually performed [4]. Thus, serial ECG during evolution survey, echocardiography, 24-h Holter monitoring of ECG, MRI with gadolinium contrast, technetium99m-sestamibi scintigraphy myocardial perfusion study, and coronary angiography can be helpful tools for diagnosis. However, all these complementary exams lack specificity for the disease [5]. Thus, in 1993, guidelines for the diagnosis of cardiac sarcoidosis have been published by the Japanese Ministry of Health and Welfare [6]. The 2 cases presented fulfill the diagnostic criteria set out in these guidelines (Table 1): cardiac abnormalities in the absence of primary heart disease with a previous diagnosis of intrathoracic sarcoidosis in the first case and Löfgren's syndrome in the second case. The clinical response to the treatment prescribed also supports our diagnosis suspicion.

**Table 1:** The Japanese Ministry of Health and Welfare 1993 guidelines for the diagnosis of cardiac sarcoidosis

**1: histological diagnosis group**	Cardiac sarcoid is confirmed then endomyocardial biopsy demonstrates epithelioid granulomas without caseating necrosis.
**2: clinical diagnosis group**	in patients with a histologic diagnosis of extracardiac sarcoidosis, cardiac sarcoidosis is suspected when: “a” and at least one of criteria “b” to “e” is present, and other etiologies such as hypertension and coronary artery disease have been excluded.
**a**	Bundle branch block, heart block of any degree, left-axis deviation, ventricular tachycardia, premature ventricular contraction, or pathological Q or ST-T change on resting or ambulatory electrocardiogram.
**b**	Abnormal wall motion, regional wall thinning, or dilation of the left ventricle.
**c**	Perfusion defect by thallium.201 or technetium-99m myocardial scintigraphy, or abnormal accumulation by gallium 67.
**d**	Abnormal intracardiac pressure, low cardiac output, or abnormal wall motion or depressed ejection fraction of the left ventricle on cardiac catheterization.
**e**	Interstitial fibrosis or more than moderate cellular infiltration over moderate grade on endomyocardial biopsy specimen, even if the findings are non-specific.

Considering the increased risk of sudden death, cardiac sarcoidosis is an indication for early treatment. Steroids should be started as soon as possible: many reports showed that, in early or middle stage disease, steroid therapy may be protective or therapeutic but may not be as effective in the late stages [7]. In both our cases, treatment was started at the middle stage of the disease and a favorable response was noted. The recommended starting dose seems to be 60 to 80 mg a day of prednisone, it can be tapered over 6 months to a dose of 10 mg a day [2]. Antiarrhythmic drugs and β blockers are also usually used in the management of cardiac sarcoidosis. To our knowledge, there have been no prospective studies evaluating the use of these medications in patients having cardiac sarcoidosis. Amiodarone can be responsible for the occurrence of pulmonary fibrosis. These drug-induced pulmonary changes, which are indistinguishable radiographically from pulmonary sarcoidosis, may threaten the patient's respiratory status. Consequently, we must carefully and constantly assess the potential benefits and risks of prescribing such medications. Regarding the treatment with ICD, it seems necessary in patients with refractory ventricular tachyarrhythmia who are at risk of sudden death in addition to antiarrhythmic drugs.

Some authors recommended ICD implantation in any patient with sarcoidosis and non-sustained ventricular tachyarrhythmia because of the high rate of recurrence of ventricular tachycardia despite medical treatment [8, 9]. As for cardiac transplantation, it is rare and remains the only possibility for younger patients with severe and stage irreversible cardiac failure or resistant VT [10]. The prognosis of cardiac sarcoidosis remains obscure: early necropsy series concluded that survival in most patients with symptomatic cardiac sarcoidosis was limited to about 2 years [3], while better outcomes were noted in other series where 5-year survival rate was 40-60%. Whether this improvement in prognosis was due to early disease recognition or a milder form of cardiac sarcoidosis versus early institution of medication remains to be determined. With progress in prevention and treatment of ventricular arrhythmias, the primary cause of death in cardiac sarcoidosis has changed from sudden death to congestive heart failure.

## Conclusion

Cardiac sarcoidosis should always be suspected in any patient who is younger than expected presenting with cardiac symptoms without evidence of primary heart disease particularly if he has already been diagnosed with sarcoidosis. To date, early diagnosis of cardiac sarcoidosis remains a challenge because clinical manifestations are non specific and the diagnostic modalities lack sensitivity and specificity in spite of recent advances. A negative endomyocardial biopsy should in no way be regarded as evidence of absence of cardiac sarcoidosis, especially if diagnostic suspicion is strong. Cardiac sarcoidosis is on occasion a medical emergency that may lead to sudden death due to the rhythm disturbances or progressive heart failure from a dilated cardiomyopathy; therefore, in addition to corticosteroid treatment, early consideration for implantation of an ICD should be a priority.
